# Burden of upper respiratory tract infections in primary care facilities and excessive antimicrobial over-prescription: A community-oriented primary care project in rural Kenya

**DOI:** 10.4102/phcfm.v13i1.3107

**Published:** 2021-11-29

**Authors:** Nelson Nyamu, Florence Mbatia, Pieter van den Hombergh, Simone Jaarsma, Felix Agoi, Jacob Shabani, Michaela Mantel, Fleur O. de Meijer

**Affiliations:** 1 Department of Family Medicine, Aga Khan University, Nairobi, Kenya; 2 Werkgroep Internationale Gezondheidszorg (WHIG), the Netherlands; 3 Department of Family Medicine, Faculty of Health, Medicine and Life Sciences, Maastricht University, Maastricht, the Netherlands; 4 Department of Population Health, Aga Khan University, Nairobi, Kenya; 5 Postgraduate Medical Education, Aga Khan University, Nairobi, Kenya

**Keywords:** upper respiratory tract infections, antimicrobials, Kenya, community oriented primary care, family medicine, antimicrobial stewardship, antimicrobial resistance

## Abstract

During their community oriented primary care (COPC) rotation in rural coastal Kenya, residents of the Family Medicine programme at the Aga Khan University–Nairobi, identified a high burden of upper respiratory tract infections (URTI) in the dispensaries with high prescription of antimicrobials (AMs) in over 80% of the patients presenting with URTI. An interactive participatory education intervention, designed based on principles of community participation and capacity building, reduced AM prescription in the under 5-year age group with 44% in the 2 weeks after the intervention, and with 18% at week 8 and 9. In the over 5-year age group, this was reduced with 18% and 8%, respectively. Key challenges for upholding AM stewardship after the intervention included the high patient workload in the clinics, difficulties in addressing patient’s concerns regarding the prognosis, inaccessibility to ingredients for home therapies, and easy availability of AMs without prescription at local chemists. Interventions addressing improper prescription at the facility level should include provision of continuous training, including communication training, for health facility staff, as well as audits on prescription practices. Collaboration with Community Health Volunteers (CHVs) can help in increasing community awareness on antimicrobial resistance (AMR). This study demonstrates the value of family physicians in clinical governance and improving the quality of care through implementation of guidelines and training. Joint action with the Kilifi county Ministry of Health and the private sector is needed to address mal-regulated access to AMs beyond health facility control.

## Introduction

Antimicrobial resistance (AMR) is a global health problem with particularly dire consequences for African countries as many first-line treatments for diseases, such as malaria, tuberculosis (TB) and human immunodeficiency virus (HIV)-related opportunistic infections, will no longer be effective and will inevitably increase the cost of care with second-line agents.^1^ The family medicine training programme at the Aga Khan University Hospital incorporates training in community-oriented primary care (COPC) at Kaloleni, situated in the rural coast of Kenya. The COPC training aims to address the main health needs of the community and to build the capacity of healthcare workers in the primary care (PC) clinics in that area.

Kaloleni has an estimated population of 192 905 from several different tribes, and most people are subsistence farmers. Health facilities serve ‘catchment’ populations in defined geographic areas within specific municipal wards. Family medicine residents work in the Kayafungo ward, which has a population of about 39 327.^2^ This ward is served by three dispensaries (Rabai, Tsangatsini and Kinarani) and one health centre (Gotani). These facilities serve as central points from which community-based primary healthcare interventions are organised by the community health unit. A community health unit consists of a community health committee and a community health officer (usually a nurse) trained to oversee a team of community health volunteers (CHVs) conducting home-visits to promote health, prevent and/or detect disease, and support treatment.^3^

## Burden of upper respiratory tract infections in Kaloleni Sub-county

The county level health information system confirmed that upper respiratory tract infections (URTI) account for more than half of all the cases seen in the health facilities. To evaluate visits for URTIs, family medicine residents (researchers N.N. and M.F.) undertook a cross-sectional evaluation of the daily patient registers. This evaluation revealed excessive over-prescription of antimicrobials (AMs) in the management of URTI in both the under-5 year age group and the over-5-year age group (including adults), with 70% – 90% of the patients receiving antibiotics (see Table 1). The use of AMs was noted to deviate from treatment guidelines, which recommend supportive therapy and the use of amoxicillin only when bacterial pharyngitis is suspected. To evaluate whether patients presenting with URTI could be managed conservatively, family medicine residents and supervising faculty participated in the care of patients during a period of 6 weeks. Although refusing AMs to patients, who had often travelled a long distance to the clinic, was an unattractive option, they were able to manage 99% of the patients seen with supportive care, using family medicine principles of communication and safety netting.

**TABLE 1:** Proportion of patients receiving antimicrobials before-and-after education.

Age group	Facility	2 week period at baseline					Week 1 and 2 post-CME					Week 8 and 9 post-CME				
		Visiting dispensary (*n*)	Patients with URTI		Patients with URTI receiving AB		Visiting dispensary (*n*)	Patients with URTI		Patients with URTI receiving AB		Visiting dispensary (*n*)	Patients with URTI		Patients with URTI receiving AB	
				
			*n*	%	*n*	%		*n*	%	*n*	%		*n*	%	*n*	%
< 5 years	Tsangatsini	304	151	50	129	85	455	394	87	119	30	307	142	46	112	79
	Gotani	336	115	34	82	71	356	161	45	77	46	191	68	35	16	24
	Both	640	266	42	211	79	811	555	68	196	35	498	210	42	128	61
≥ 5 years	Tsangatsini	723	301	42	268	89	502	180	36	135	75	437	99	23	88	89
	Gotani	1011	273	27	236	86	827	140	17	89	64	491	82	17	57	70
	Both	1734	574	33	504	88	1329	320	24	224	70	928	181	20	145	80

**Total**		**3014**	**840**	**35**	**715**	**85**	**2951**	**875**	**41**	**420**	**48**	**1924**	**391**	**27**	**273**	**70**

## Interactive educational intervention

An interactive educational intervention with the health facility staff and CHVs was done to highlight the burden of URTIs as the most common presenting ailment in the health facilities. The purpose of the intervention was to increase adherence to Integrated Management of Childhood Illnesses (IMCI) guidelines for the children under 5-years and adherence to the Kenyan Government Clinical Guidelines for the over-5-year age group. Educating CHVs was intended to motivate them to spread knowledge on sensible use of AMs during their household visits. The educational sessions were fully interactive, exploring understanding of the staff on AMR, factors influencing the high number of clinical visits for URTIs, and the reasons for the high antimicrobial (AM) usage. A presentation on the management of URTIs, based on the Kenyan guidelines was made, with a discussion on evidence-based management. A post-intervention review of the registers was done over a 2-week period directly after the intervention and again 8–9 weeks later to evaluate the effect on prescribing. Findings are presented in Table 1.

**FIGURE 1:**
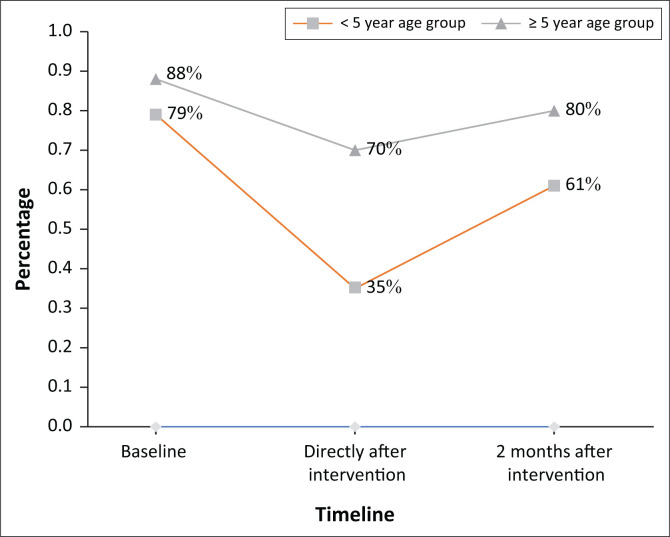
Percentage of patients presenting with upper respiratory tract infections at Gotani and Tsangatsini facilities who were treated with antimicrobials in the < 5-year and ≥ 5-year age groups.

Antimicrobial prescription in the under 5-year age group was reduced by 44% in the 2 weeks following the intervention, and by 18% at weeks 8–9. In the over 5-year age group, prescribing was reduced by 18% and 8%, respectively, (see Figure 1).

## Reflections on this intervention

This COPC exercise focused on the improper prescription of AMs in Kenyan rural health facilities, contributing to AMR. Although we did not compare our data with a control group (controlling for potential other factors influencing antibiotic prescription), the findings suggest that education and participatory discussion that sought to influence the knowledge and prescribing behaviour of health personnel, had a positive effect on antibiotic stewardship and resulted in a reduction in AM prescription post-intervention. Nevertheless, the positive effect of the intervention was less noticeable after 2 months.

A multifactorial approach is required, both when evaluating driving forces behind AM over prescription, and when implementing efforts for control.^4^ Factors known to influence prescribing practices for URTI in PC include varied knowledge on AMR amongst lower level healthcare workers working at first line facilities, absence of diagnostic tests to guide clinical decision-making, and shortage of healthcare personnel.^4,5,6^ Nurses and clinical officers working in our facilities were knowledgeable on the consequences of over prescription and the treatment guidelines for URTI in the under 5-year age group, but often unfamiliar with guidelines for the over 5-year population, which may have contributed to lower guideline-adherence in that age group. Despite improved knowledge after the education, they reportedly struggled with a high workload, which prevented them from performing an adequate history and physical examination. In the absence of a definitive diagnosis, they often considered the financial burden and time involved for a patient that would have to return for a second consultation in case of no improvement. Mothers with children presenting with only a runny nose seemed to be knowledgeable on the more severe features of pneumonia, such as fast breathing and lethargy. Nevertheless, healthcare staff faced challenges in explaining the self-limiting nature of the common cold and perceived dissatisfaction with care provided when AMs were withheld. An aggravating factor was the unavailability of recommended home remedies such as lemon, turmeric and honey. This resulted in a prescription-guided approach to care where antibiotics were prescribed to cover for any undiagnosed bacterial infections.

Community health volunteers reported that patients often failed to finish treatments, shared medication within families and often relied on non-prescription based AMs from local chemists and private clinics. Poorly regulated access to AMs is known to be a problem in many low- and middle-income countries, which further increases AM misuse while hampering any AM stewardship effort within healthcare facilities.^6^

Further interventions to reduce AM misuse should therefore be directed both at facility and community level. Health register audits and continuous medical education (CMEs), as performed by our family medicine residents, should be offered more regularly to uphold evidence-based practice and stimulate antibiotic stewardship. Personal feedback to healthcare staff should be given to improve prescribing, but is time-consuming and might be a challenge with limited resources.^5^ Furthermore, communication skills training could aid health facility staff to better address patient concerns regarding prognosis, benefits, and harms of antibiotic treatment.^6^ The CHVs were invited to our educational intervention as they form an important link to the community and could help reduce the visits to healthcare facilities by informing households on the self-limiting nature of a common cold. Hygiene and ventilation to prevent household spread of URTIs should also be emphasised. To address unregulated access to AMs, a multi-sectoral approach looping in the private facilities and local dispensaries would be needed. The huge economic costs that result from over-prescribing and subsequent AMR should guide future interventions on county level in conjunction with family medicine initiatives.

## Contribution of family medicine

The family medicine team was able to highlight the burden of URTI in the PC clinics as well as the factors leading to AM over prescription. This study demonstrates the value of family physicians in clinical governance and improving the quality of care through implementation of guidelines and training. By working in the clinics and doing home visits, family medicine residents had a good understanding of the workload experienced by the health facility staff. This was beneficial in the discussion around judicious use of AM. Inviting both HCWs and CHVs to the capacity building exercise allowed for integration of community and PC approaches. The family medicine programme at the Aga Khan University is planning to offer ongoing training on AMR and communication skills by having subsequent groups of residents follow up on COPC activities previously started by their peers. Key family medicine principles of providing comprehensive, evidence based and cost-effective care will be key in future CMEs.

## Conclusion

The conservative management of URTIs offers an opportunity to reduce the workload in PC facilities. This would allow healthcare workers to focus on other conditions in addition to reducing the expenditure in the management of URTI. More importantly, it could contribute to protecting the community from the severe consequences of AMR. Family physicians through COPC principles could play a key role in spearheading AM stewardship in rural health facilities and in increasing community awareness on AMR. Collaborative interventions on county and national level are needed to regulate sales of AMs and further reducing misuse beyond health facility control.
